# 466. Interrupted Time-Series and Survival Analysis of Adult Hospitalized Pneumococcal Disease from 15-Year Nationwide Surveillance: Evidence Supporting Vaccine Inclusion in National Immunization Programs of Resource-Limited Countries

**DOI:** 10.1093/ofid/ofaf695.157

**Published:** 2026-01-11

**Authors:** Thundon Ngamprasertchai, Jintana Srisompong, Rattagan Kajeekul

**Affiliations:** Faculty of Tropical Medicine, Mahidol University, Bangkok, Krung Thep, Thailand; Suratthani Hospital, Bangkok, Krung Thep, Thailand; Maharat Nakhon Ratchasima Hospital, Nakhon Ratchasima, Nakhon Ratchasima, Thailand

## Abstract

**Background:**

Thailand has yet to include any pneumococcal vaccines in its National Immunization Program (NIP) as of 2025, contributing to low vaccine uptake and a persistently high burden of pneumococcal diseases. This study aims to update the incidence of pneumococcal diseases in Thai hospitalized adults using time-series analysis of data from sentinel hospitals across the country to generate evidence supporting its inclusion in the NIP.

**Figure:**
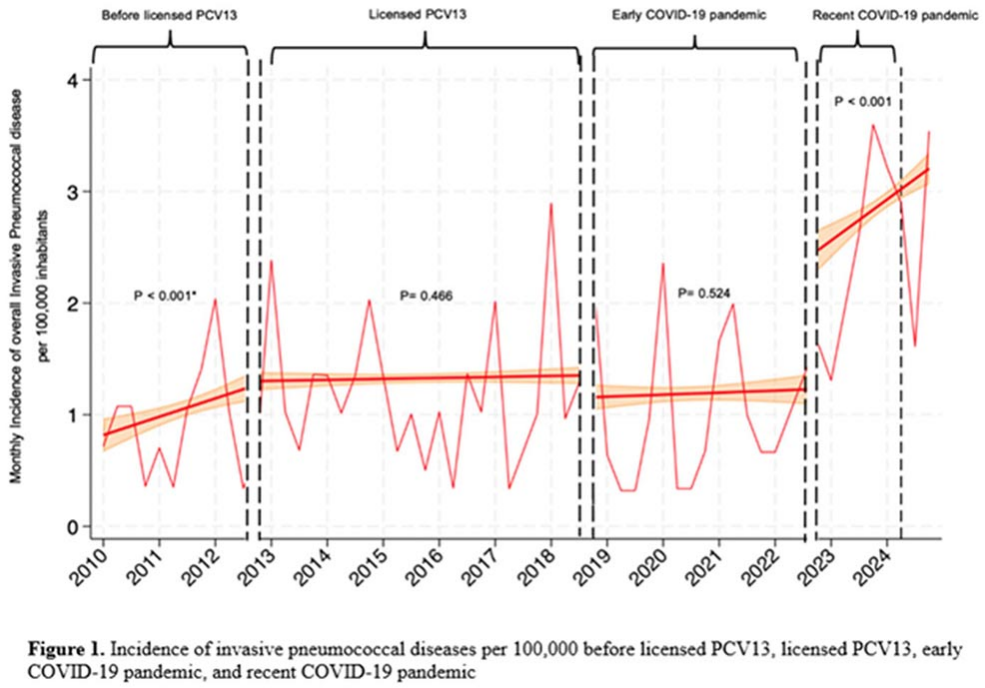

**Figure:**
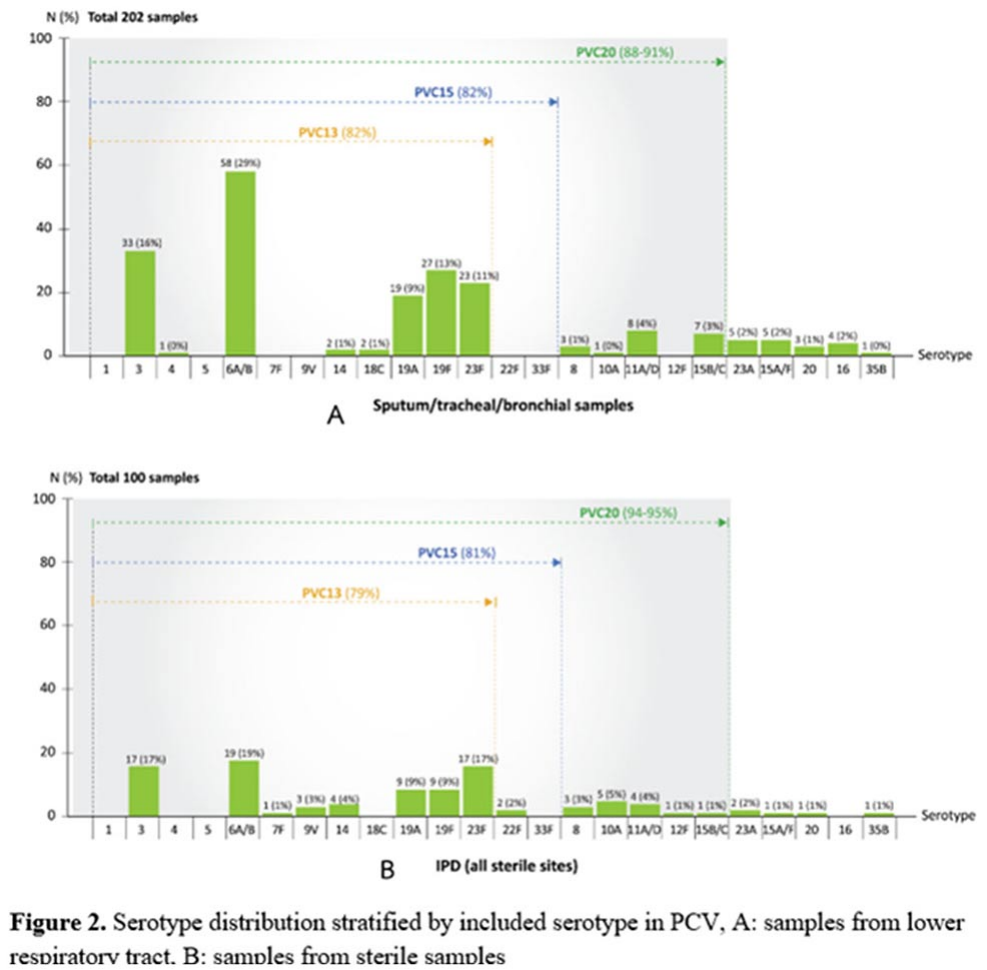

**Methods:**

A quasi-experimental, interrupted time-series analysis was conducted using sentinel-based, prospective surveillance of adults aged ≥18 years hospitalized with invasive pneumococcal disease (IPD) or pneumococcal pneumonia (PP) in Thailand from 2010 to 2024. Serotype distribution was assessed through parallel prospective surveillance. The study period was divided into four phases based on PCV13 licensing and the COVID-19 pandemic. Segmented linear regression with autoregressive adjustment was used for time-series analysis.

**Results:**

A total of 2,069 adults hospitalized with pneumococcal diseases were enrolled, including 61.0% aged >65 years, 15.2% aged 50–65 years, and 23.5% aged 18–50 years. PP accounted for 72.8% of cases and IPD for 27.2%. IPD incidence was stable from 2010 to 2022 (1.0–1.45 per 100,000; p = 0.47) but rose significantly during the recent COVID-19 pandemic (3.1 per 100,000; p < 0.001) (Figure 1), similar trends in PP. The highest incidence was among those aged >65 years (9.2 per 100,000). Overall in-hospital mortality was 23.4%, with higher mortality in IPD cases (p < 0.01). Vaccine serotype coverage for PP was 82% for PCV13/15 and 88–91% for PCV20; for IPD, coverage was 79%, 81%, and 94–95%, respectively. The predominant serotypes were 6A/B and 3 (Figure 2).

**Conclusion:**

The burden of pneumococcal diseases in Thailand remains substantial, with a notable increase observed during the COVID-19 pandemic. Adults aged >65 years were the most affected group. The majority of disease-causing serotypes remained vaccine-type strains. Given that PCV13 and PCV15 provide coverage for approximately 80% of circulating serotypes, their inclusion in the Thai adult NIP should be prioritized to reduce the disease burden.

**Disclosures:**

All Authors: No reported disclosures

